# Durability of pneumatic dilation monotherapy in treatment-naive achalasia patients

**DOI:** 10.1186/s12876-019-1104-z

**Published:** 2019-11-11

**Authors:** Abid T. Javed, Kevin Batte, Mohamed Khalaf, Mustafa Abdul-Hussein, Pooja S. Elias, Donald O. Castell

**Affiliations:** 1 Department of Internal Medicine, Medical University of South Carolina. Charleston, SC 787 Navigators Run, Mount Pleasant, SC 29464 USA; 2 Department of Internal Medicine, Division of Gastroenterology and Hepatology. Medical University of South Carolina. Charleston, SC 114 Doughty St, 2nd floor, Charleston, SC 29425 USA

**Keywords:** Achalasia, Pneumatic dilation, Esophagus, Manometry, Outcomes

## Abstract

**Background:**

Pneumatic dilation (PD) is often billed as a “short term” treatment for achalasia but anecdotally can last years. This study sought to explore how long a single pneumatic dilation may induce symptom remission in a treatment-naïve achalasia patient.

**Methods:**

A single center, retrospective chart review of patients with an ICD-9 or − 10 code of achalasia between 2005 and 2017 was performed. Treatment naïve patients with manometric diagnosis of primary achalasia were included. Outcomes (success or failure); single vs multiple PD; age; and estimated duration of effect were evaluated. Each patient underwent a single PD unless re-intervention was required for relapse.

**Results:**

83 patients (52% female, median 51.6 ± 3.6 years) were included. 43% underwent 2 PD and 13% underwent 3 PD. There was no significant relation between age, gender, and number of PDs. After 1 PD, 87.5% of patients reported > 1 year of symptom remission. 80.5% of relapsed patients reported success after a 2nd dilation. 1 PD was more likely to result in success than multiple PDs (*p* < 0.001). The measured median duration of remission after 1 PD was 4.23 years, and for 2 PDs, 3.71 years. The median estimated remission time after 1 PD was 8.5 years (CI 7.3–9.7, *p* = 0.03).

**Conclusions:**

PD is a safe, durable treatment for achalasia. A single PD is likely to last years. A second PD, if required, also has a high likelihood of success.

## Background

Achalasia is a disorder characterized by impaired esophageal motility due to a) loss of peristaltic activity and b) failure of the lower esophageal sphincter (LES) to relax during swallowing [1–4]. It is a relatively rare disorder, with a cited incidence of 2–3 per 100,000 [5, 6] and a prevalence of 1 per 10,000 [7].

Achalasia usually presents as a combination of dysphagia, chest pain, weight loss, and regurgitation. It enters the differential after structural causes of dysphagia have been ruled out [8–11]. Esophageal motility testing and barium esophagram are complementary in support of the correct diagnosis. The gold standard for achalasia diagnosis is high resolution manometry. This tool was integral in the creation of the newest Chicago Classification system regarding guidelines for dysmotility diagnosis [9–12].

Analysis of achalasia treatment outcomes is an ongoing area of research. Pre-treatment variables such as age, gender, LES pressure, LES length, and esophagus diameter seem to influence success or failure [13, 14]. Outcomes based on achalasia type are an emerging focus of research [11, 12, 15]. Achalasia type III is suspected of having the least favorable outcome with pneumatic dilation (PD) [15–18].

The success rate of pneumatic dilation is the subject of constant re-evaluation, especially as it compares to laparoscopic Heller myotomy (LHM). Numerous studies have found pneumatic dilation to be non-inferior compared to LHM [19–25]. The most recent European multicenter trial found no significant difference between the success rate of LHM and PD (84% vs. 82%, respectively), with a 25% re-dilation rate for patients undergoing PD [23]. Another study found a 37% remission rate in patients who underwent one PD compared to 86% using a graded dilation protocol [24]. When comparing complication rates between PD and LHM, the rate of perforation, most commonly feared complication, is reported to be ~ 2% in PD and 2–10% in LHM [10, 19, 25]. Comparisons of traditional treatment modalities relative to per-oral endoscopic myotomy (POEM) is another area of emerging research.

Our aims were to survey prevalence of single vs. multiple PDs; to assess duration of remission in patients with achalasia treated with PD as monotherapy; and to evaluate outcomes of patients requiring single vs. multiple PDs. These aims were borne out of our senior author’s anecdotal experience that a single pneumatic dilation without planned or graded re-dilation can produce symptom relief lasting greater than one year.

## Methods

Permission was obtained from the Institutional Review Board at the Medical University of South Carolina in February 2017 to carry out this study. Consecutive patients treated at our institution from January 2005 through December 2017 with an ICD-9 code of 530.0 or ICD-10 code of K22.0 were identified for retrospective chart review.

### Inclusion criteria and stratification

Patients with a diagnosis of achalasia confirmed via conventional manometry or HRM who underwent pneumatic dilation as a primary means of treatment were included in the initial sample. Patients were retrospectively placed independently into three groups based on number of pneumatic dilations. Those who underwent one PD were grouped as “PDx1”; those who underwent two PDs were grouped as “PDx2”; and those who underwent three PD were grouped as “PDx3”. Patients were assigned independently, i.e. if a patient underwent two PDs within the time period, the patient’s data was only analyzed once in the PDx2 group.

### Exclusion criteria

Exclusion criteria included incorrect coding, prior anatomy altering surgery, concomitant esophageal disease, or prior non-PD treatment. Patients with secondary achalasia (malignancy, infectious, post-surgery, infiltrative diseases, etc.) were also excluded, as were those in whom sufficient follow up data of at least 12 months was unavailable.

### Intervention and technique

The technique employed has been used historically by our senior author (DOC) for more than two decades. All patients underwent pneumatic dilation with a Rigiflex pneumatic dilator (Boston Scientific Inc., Marlborough, Massachusetts, USA). Patients initially were treated with a 30 mm balloon. Subsequent dilations were performed using a 35 mm balloon. The senior author was present for every dilation ensuring uniform technique. After endoscopic examination, guidewire was placed across the gastro-esophageal junction and the balloon inserted. The balloon was inflated to 30 or 35 mm and held at 10 psi for 15 s, or until obliteration of the waist was confirmed. Immediately post-dilation, while the patient was sedated, a post-procedural Gastrografin study was completed via nasogastric tube placed into the esophagus. Dilations were performed under fluoroscopic guidance, and post-procedural images were obtained to evaluate for extravasation outside the esophageal lumen.

### Study outcomes: success and failure

After PD, patients followed up in clinic at 1, 6, and 12 month intervals, and subsequently every 2 years, to assess symptom relapse. Success was defined as symptom remission for greater than one year after pneumatic dilation by patient report and by Eckardt score less than 3 at one year. Patients were defined as failures if symptoms recurred within one year. If patients underwent multiple pneumatic dilations, they were considered failures if the most recent pneumatic dilation failed to relieve symptoms for greater than one year (or improved Eckardt score as above). Duration/follow up time (in days) was measured as date of PD to date of most recent clinic visit.

### Statistical analysis

Statistical analyses were performed using MedCalc for Windows, version 15.0 (MedCalc Software Ostend, Belgium). Quantitative variables were summarized using counts (n), means, medians, ranges, or standard deviations where applicable. Qualitative data were expressed in frequencies (n) or percentages (%). The age of patients pursuing one vs multiple PDs was analyzed using an independent t test. The age at the time of the repeat dilation was analyzed using a Mann Whitney U test as these data did not meet assumptions needed for independent t testing. PD outcome, duration of effect, differences in one vs multiple PDs, and impact of age and gender were analyzed using Fisher Exact tests.

Pneumatic dilation success was assessed by analyzing symptom presence at time of follow up, as well as by using Kaplan-Meier survival analyses to indicate estimated probability of symptom relapse. A Kaplan-Meier curve was also used to estimate probability of symptom relapse in patients greater than 40 years old compared with those less than 40, as previously examined by Eckardt et al. [27]. There results were expressed as estimated probability of remission. A Spearman rank order correlation test was used to assess correlation between pneumatic dilation and adverse events.

All results were considered significant if *p* ≤ 0.05.

## Results

**Fig. 1:**
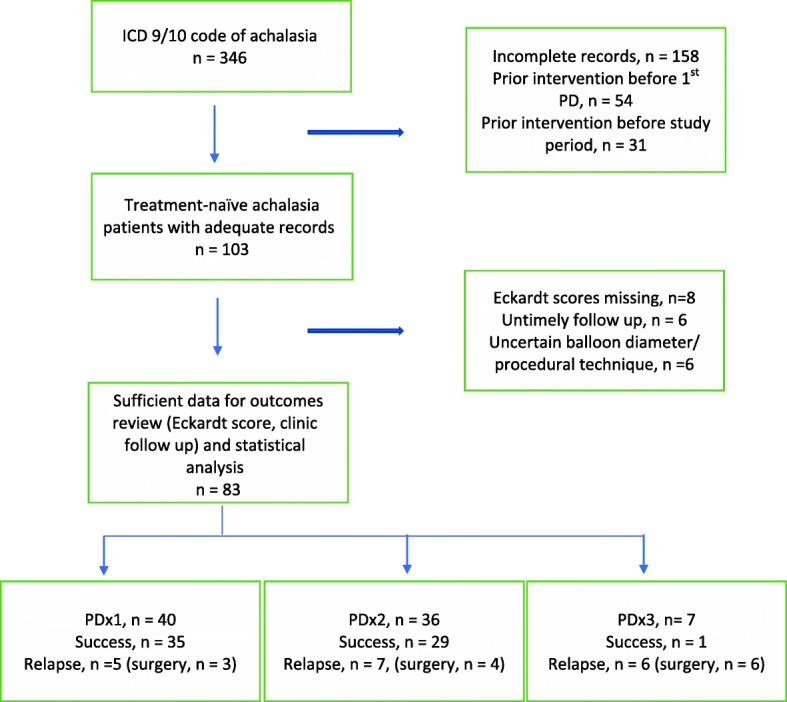
Flowchart of patient inclusion and outcome

346 patients were identified with an ICD-9 or ICD-10 code of achalasia. Records were reviewed for these patients to identify those who underwent pneumatic dilation. After application of the exclusion criteria, there was sufficient documentation to obtain initial PD data for 103 patients. We were able to verify outcomes (symptom relapse or remission) on 83 patients. Of 20 patients for whom outcomes data was unverifiable, 16 underwent one PD, 3 underwent two PDs, and 1 underwent three PDs (Fig. 1.).

Of 83 patients, 43 (51.8%) were female. 40/83 (48.1%) underwent one PD (PDx1), 36/83 (43.4%) underwent two PDs (PDx2), and 7/83 (8.3%) underwent three PDs (PDx3). The median ages (at first dilation) by number of PDs were: PDx1, 58.1 years (range 23–81); PDx2, 51.7 (19–86); and PDx3, 39.0 (20–73), respectively. At the time of first dilation, those who pursued 2 or more PDs were significantly younger (49.6 ± 5.4 years) than those who pursued one PD (57.4 ± 3.5 years), (*p* = 0.03). Patients who underwent three dilations were significantly younger (39 ± 11 years) than those who underwent two dilations (52.9 ± 5.4 years) at time of second dilation (*p* = 0.04). The median age of patients at their third pneumatic dilation was 37.4 years (range 24–77). The median time interval between PDs in patients who underwent two PDs was 654.6 days (range 14–2335 days). In patients who underwent 3 PDs, the median time between first and second PDs was 119.9 days (range 19–210 days), and 804.9 days (range 49–1841 days) between the second and third PDs. There is a significant difference in intervals between first and second dilation between the PD2 and PD3 group (*p* = 0.047).

### Outcomes of pneumatic dilation

**Fig. 2:**
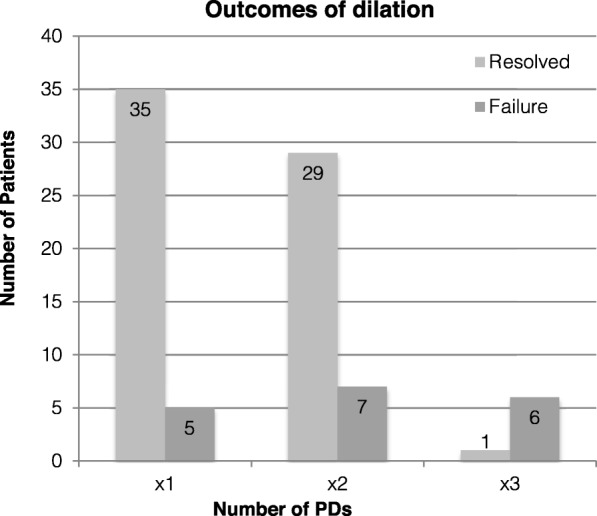
Outcomes of PD (resolution or failure) retrospectively sorted by number of PDs

Outcomes data were collected on 83 patients. 40/83 (48.1%) patients underwent a single pneumatic dilation. 35/40 (87.5%) achieved symptom remission for at least one year. 3/40 (7.5%) were referred for surgery due to symptom relapse, and 2/40 (5%) relapsed and chose not to pursue further interventional treatment (Fig. 2.). Of 36 patients who underwent two dilations, 29/36 (80.6%) achieved remission, 4/36 (11.1%) were referred for surgery, and 3/36 (8.3%) failed PD and did not seek further treatment. Only 1/7 (14.5%) patients who underwent three dilations experienced long term symptom resolution; 5/7 (71%) pursued a surgical alternative after their third dilation. The cumulative re-dilation rate was 59.4% (cumulative number of repeat PDs divided by total patients). The cumulative re-dilation success rate (success of subsequent PD divided by number of instances of re-dilation) was 69.7% (30/43). When comparing one PD to multiple PDs, there was no significant difference between the number of successes and the number of failures (*p* = 0.06). Overall, a majority of patients found success with PD. The mean Eckardt score before and after a successful pneumatic dilation was 7.3 (SD 2.9) to 2.2 (SD 1.6), respectively, compared to 6.6 (SD 2.2) to 6.4 (SD 2.3) in those who failed pneumatic dilation.

### Duration of pneumatic dilation effect

**Fig. 3:**
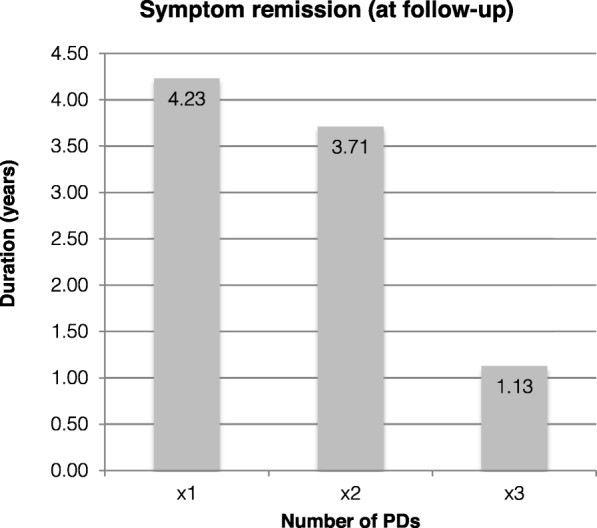
Time interval in mean years between date of PD and most recent follow-up of patients who experienced symptom resolution (i.e. measured duration of success)

**Fig. 4:**
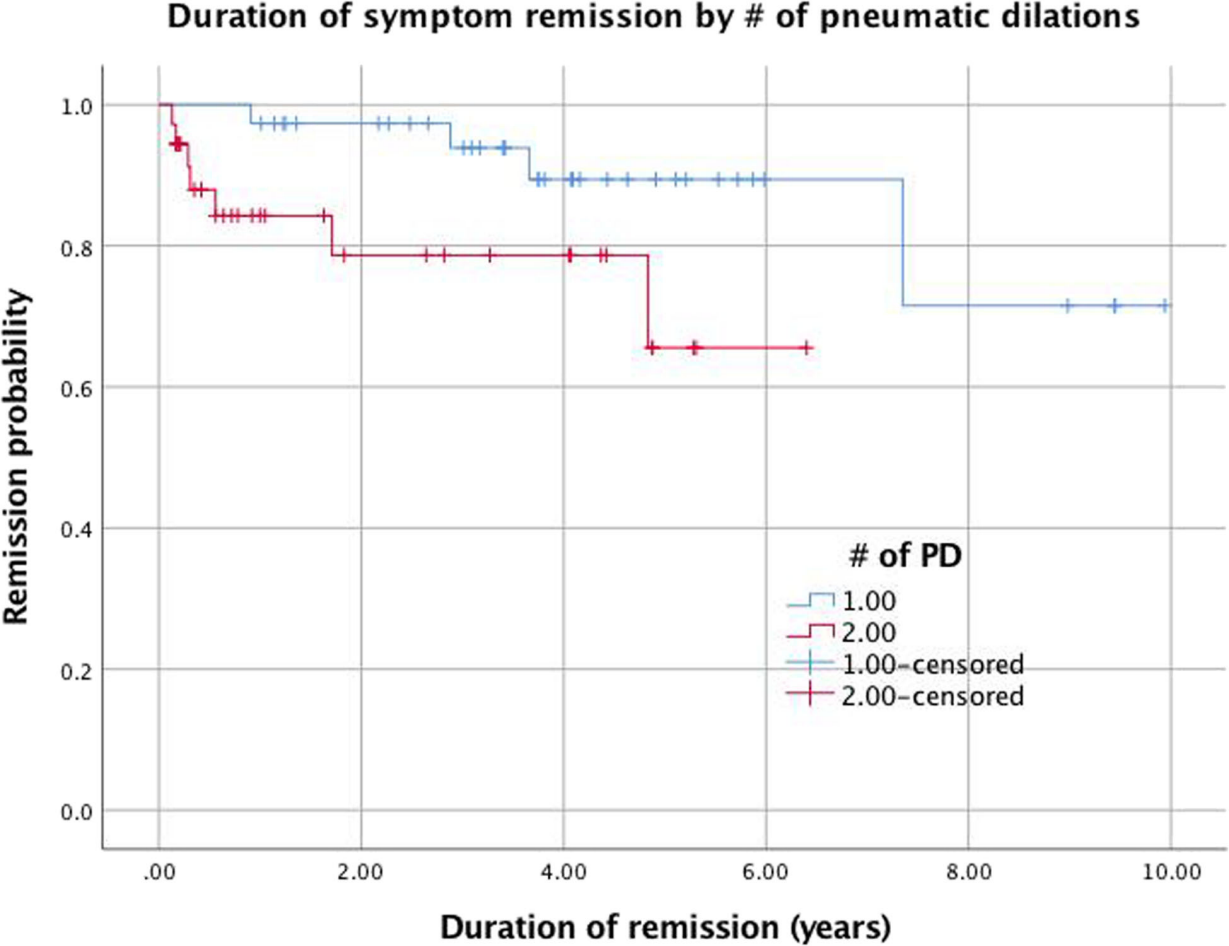
Kaplan-Meier recurrence-free survival of 83 patients after pneumatic dilation (PDx1 vs PDx2)

An important aspect of this study was assessing how long the effect of a pneumatic dilation lasted. Measuring this outcome was attempted in two ways: a) by recording the time between the intervention and date of most recent follow up (Fig. 3.), and b) by using Kaplan-Meier graphs to extrapolate how long patients remain in remission post-PD (Fig. 4.).

Duration of outcome was classified by number of PDs. For patients with successful outcome as defined in Methods, the median duration of remission with an endpoint defined by date of last follow up was: for one PD, 1544.3 days (4.23 years, range 367–3267 days); for two PDs, 1313.7 days (3.71 years, range 386–2768 days); and for three PDs (*n* = 1), the duration was 411 days, or 1.13 years.

Amongst patients who relapsed and pursued surgery, the median follow up time from PD to clinic was 1400.7 days (3.83 years, range 186–2681 days) for PDx1 patients; 2036.7 days for PDx2 (5.58 years, range 499–4041 days); and 1220.8 days (3.34 years, range 268–2297 days) for PDx3. For patients who failed PD (relapse within one year) but did not pursue further interventional treatment during the study period, the median follow up period was 692 days (1.90 years, range 333–1051 days) for patients who had one PD, and 943 days (2.58 years, range 690–1240) for patients who had two PDs.

There was no significant difference in follow up time between successful PDs and failed PDs (*p* = 0.65). Similarly, there was no significant difference in the duration of symptom remission between one vs multiple PDs (*p* = 0.19).

Duration of remission (i.e, duration of symptom resolution) was also evaluated by Kaplan-Meier survival analysis. This provided a cumulative probability of remission as well as an estimate of median survival time. As seen in Fig. 4, the cumulative probability of remission in the PDx1 group was ~ 87% at five years. For the PDx2 group, the cumulative remission percentage at five years was ~ 78%. The PDx3 group had a much poorer estimated percent remission of ~ 20% at 5 years. The probability of remission was significantly higher in those who underwent only one PD compared to multiple (*p* < 0.005). The median remission time was estimated at 8.5 years (95% CI 7.3–9.7) for the PDx1 group. For the PDx2 group, the estimated median remission time was 5.0 years (4.0–5.9). The PDx3 group had an estimated median remission time of 3.4 years (1.9–4.9). Here, there is a significant difference between the median remission of the PDx1 and PDx2 groups compared to the PDx3 group (*p* = 0.03).

### Impact of age and gender on outcome

**Fig. 5:**
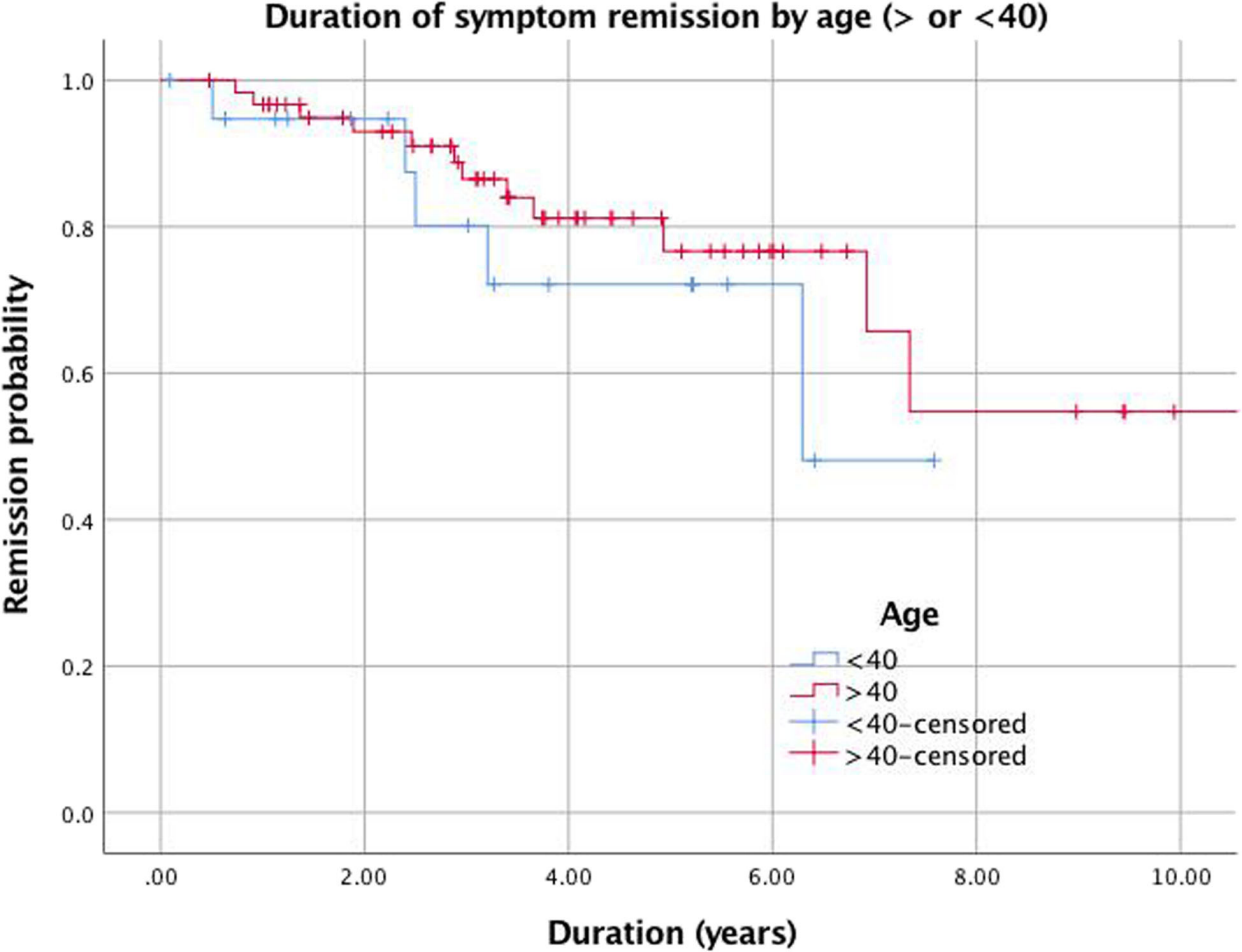
Kaplan-Meier recurrence-free survival of 83 patients after pneumatic dilation (age > 40 years vs < 40 years)

Impact of age on outcome of pneumatic dilation was examined using Kaplan Meier survival analysis. Patients were stratified in groups of < 40 and > 40 When excluding the PDx3 group, which had a median age of just over 40, younger patients achieved a longer length of remission after 3.5 years, though these results were not statistically significant (*p* = 0.48) **(**Fig. 5.**).** When including the PDx3 group, where most patients were younger than 40, patients > 40 had a slight remission percentage advantage, though these results also were not statistically significant (*p* = 0.27). When comparing success and failure of patients < 40 to patients > 40, there was no significant difference (*p* = 0.33). There was also no significant difference when comparing success and failure by gender (*p* = 0.26).

### Adverse events

There was one report of post-procedural chest pain that required an emergency room visit; this resolved with conservative treatment. There were two instances of micro-perforations noted on post-dilation barium swallow, a rate of 1.9%. Both resolved with conservative management and did not require surgery. One case occurred in a patient who underwent two PDs before pursuing surgery; the other occurred in a patient who underwent only one PD and who achieved long-term success. There were three cases (3.6%) reported of post procedural gastroesophageal reflux all improving with medical therapy. By Spearman correlation analysis, there was not a significant correlation between PD and complication events (*p* = 0.14).

### Manometry

Of those 23 patients where manometric subtype was available prior to the first PD, the following were noted. For PDx1 (*n* = 12), there were 7 type 1 achalasia patient and 5 type II; for PDx2 (*n* = 10), 7 type 1 and 3 type 2; and for PDx3 (n = 1), there was 1 type III achalasia patient. Statistical analysis was not performed here as not all types were represented amongst the three groups.

## Discussion

Numerous studies have attempted to define expectations regarding the duration of symptom remission induced by pneumatic dilation. We examined our institution’s achalasia patients who were treatment naïve to learn how long one could expect a pneumatic dilation to last. Our interest was borne out of our senior author’s experience that some patients achieve many years’ success with two dilations or less. In fact, the single longest symptom free period of all patients who were included in our study was greater than 11 years after only one PD. For those who had two PDs, the longest duration of remission at follow up was 7.33 years.

Current interventions for achalasia include pneumatic dilation (PD), laparoscopic Heller myotomy (LHM), per-oral endoscopic myotomy (POEM), and Botox injections [18]. PD and LHM represent the most prevalent interventions, while POEM is the most recent innovation in achalasia treatment. LHM was initially thought to be the most successful treatment, but recent studies have shown that PD approaches the success rate of LHM [21, 33–36], especially when accounting for manometric subtype [13, 14, 17, 22]. However, studies differ in their methodologies, type of manometry (conventional vs HRM), and techniques of LHM, PD, and POEM. Additionally, some studies fail to account that many patients undergo various combinations of multiple procedures [17, 18]. No recent study has analyzed treatment naive patients who have undergone pneumatic dilation exclusively using Rigiflex balloons 30 or 35 mm in size. While short term success, generally defined as symptom improvement > 6 months or as a reduction in Eckardt score to less than or equal to 3, of all therapies is well documented [4, 9, 10, 16, 19], the duration of treatment response and long-term outcomes are not well-known and harder to predict, especially when accounting for uniform technique and procedure.

PD technique has evolved over time. In one of the first modern studies to evaluate PD efficacy, PD was performed with handmade balloons before 1994; the authors then switched to the early Rigiflex balloons and continued data collection [26]. Eckardt’s 2004 study used Brown McHardy dilators in initial and subsequent dilations, introducing another variable into the equation, as these dilators are uniformly 35 mm in size [27].Planned, graded re-dilation, where the patient returns in 4–6 weeks despite change in symptoms for re-dilation with a larger balloon (usually 35 mm), is another technique that has been previously employed [28–31]. The use of 40 mm balloons is sporadic amongst those who perform pneumatic dilations, and our institution no longer uses them. Elias and Castell posit that inflating the balloon until the esophageal “waist” has been obliterated is a better dilation technique. This was the approach used uniformly in our study. Several other studies including one by Gideon et al. have noted that shorter distension times achieve similar success rates [32, 33]. While no comparative studies comparing dilation techniques have been performed, our data suggest that a single pneumatic dilation inflated until the waist is obliterated is a viable procedural standard.

Our data show that it is reasonable to expect at least one year of symptom resolution following pneumatic dilation. Though differing in procedural technique and approach to symptom relapse, our results follow a 2010 Belgian study with similar conclusions, noting that 66% of their patients were symptom free between 5 and 7 years after a single pneumatic dilation [37]. This is the largest European study to analyze a similar question as ours. The re-dilation success rate is also favorable (80.6%). About a third of our patient population ultimately pursued repeat dilation, compared to a previously reported figure of 25% by the European multicenter trial [23, 24]. However, our figure is much lower than the 52% rate of repeat intervention reported by Lopushinsky et al. [38]. We have demonstrated that planned graded balloon dilation is not necessary to improve symptoms unless there is a clear recrudescence of disease. Though this method does work [19–21], our data clearly indicate that a single PD may be sufficient to produce lasting results. Again, we employ a single pneumatic dilation based on our senior author’s anecdotal experience and practice for > 20 years, prior to the publication of the studies above. While we acknowledge the practice employed by many practioners, we sought in this paper to demonstrate a different yet effective approach to the treatment of achalasia.

Several studies indicate that younger patients, including males, may be more likely to relapse, are referred for surgery earlier, and may have a predilection for type III achalasia [13, 15–18, 24, 37] Our data support this trend, especially when looking at those who pursued three pneumatic dilations. Of these seven, the majority were males less than 40 who all ended up pursuing surgery. Though the sample of patients who underwent 3 PDs was small (*n* = 7), we surmise that advancements in pneumatic dilation, understanding of achalasia pathophysiology, and refinement in other treatment modalities have reduced the utility of pursuing more than two pneumatic dilations.

Our study had several limitations. Classically, retrospective studies are susceptible to attrition and sampling biases. Our small sample size diminished our statistical power. We were hampered by a change in medical records at our institution that resulted in many potential subjects being excluded due to lack of records. We were unable to include achalasia type as a variable as early patients did not undergo HRM. However, of the 7 who underwent 3 pneumatic dilations, 5 were retrospectively read as having type III achalasia, supporting new data that type III achalasia is less amenable to pneumatic dilation. Future studies should expand on outcome by achalasia type, as well as comparisons of monotherapy for achalasia, while clearly delineating patient population, diagnostic and therapeutic criteria, and procedural technique to eliminate confounders of treatment outcome.

## Conclusions

We conclude that the majority of treatment naïve achalasia patients achieve symptom remission for greater than one year after pneumatic dilation. Based on Kaplan Meier analysis, it is not unreasonable to expect five years of symptom remission in most patients. Overall, pneumatic dilation remains a safe procedure that may be more durable than generally believed.

## Data Availability

Data will be uploaded as a supplementary file. It has not been recorded in a public repository.

## Abbreviations

HRM: high resolution manometry
LES: Lower esophageal sphincter
LHM: laparoscopic Heller myotomy
PD: pneumatic dilation
POEM: per oral endoscopic myotomy
